# It’s all connected! Multivariate pattern analysis of inter-network connectivity distinguishes between reappraisal and passive viewing of emotional scenes

**DOI:** 10.1093/cercor/bhaf161

**Published:** 2025-06-25

**Authors:** Scarlett Horner, Thomas Rawliuk, Ryan M Ferstl, Andrew L Lyons, Janeen Martin, Diana J Gorbet, W Dale Stevens, Steven G Greening

**Affiliations:** Brain & Cognitive Sciences, Dept. of Psychology, University of Manitoba, 190 Dysart Rd., Winnipeg, MB R3T 2N2, Canada; Brain & Cognitive Sciences, Dept. of Psychology, University of Manitoba, 190 Dysart Rd., Winnipeg, MB R3T 2N2, Canada; Brain & Cognitive Sciences, Dept. of Psychology, University of Manitoba, 190 Dysart Rd., Winnipeg, MB R3T 2N2, Canada; Brain & Cognitive Sciences, Dept. of Psychology, University of Manitoba, 190 Dysart Rd., Winnipeg, MB R3T 2N2, Canada; Brain & Cognitive Sciences, Dept. of Psychology, University of Manitoba, 190 Dysart Rd., Winnipeg, MB R3T 2N2, Canada; York MRI Facility, York University, 4700 Keele St., Toronto, ON M3J 1P3, Canada; York MRI Facility, York University, 4700 Keele St., Toronto, ON M3J 1P3, Canada; Department of Psychology, York University, Behavioural Science Building, 4700 Keele St., Toronto, ON M3J 1P3, Canada; Brain & Cognitive Sciences, Dept. of Psychology, University of Manitoba, 190 Dysart Rd., Winnipeg, MB R3T 2N2, Canada; Centre on Aging, University of Manitoba, 183 Dafoe Rd., Winnipeg, MB R3T 2N2, Canada

**Keywords:** attention control network (ACN), default mode network (DMN), functional connectivity, multivariate pattern analysis, reappraisal

## Abstract

Down-regulation using reappraisal is often associated with negative connectivity between prefrontal areas such as the dorsolateral prefrontal cortex (dlPFC) and areas associated with emotion such as the insula and amygdala, though a network perspective is often lacking in emotion regulation research. Whereas the dlPFC is associated with the attentional control network (ACN), the insula and amygdala are associated with the salience and limbic networks, respectively. The default mode network (DMN), including the ventromedial PFC, also contributes to emotion regulation. The present study sought to determine if inter-network functional connectivity can dissociate reappraising from passively viewing a negative image using multivariate pattern analysis (MVPA). Thirty-one participants completed a functional magnetic resonance imaging task in which they reappraised and viewed negative images. Behavioral and skin conductance response results indicated that reappraisal was associated with reductions in negative affect compared to viewing. The univariate connectivity analysis revealed that connections between aspects of the DMN and ACN differed between reappraising versus viewing negative images. Notably, the inter-network connectivity MVPA results demonstrated that whether one was reappraising versus viewing an image could be predicted better than chance, with several connections reliably contributing to the model, including those between ACN and DMN.

## Introduction

Reappraisal is a form of emotion regulation in which one changes one’s thoughts or interpretations of an emotional stimulus to change or maintain their emotional state (Gross 1998, 2002). The Selection, Optimization, and Compensation with Emotion Regulation (SOC-ER) model states that the success of emotion regulation, including reappraisal, hinges on available internal resources (Urry and Gross 2010; Opitz et al. 2012). Internal resources is an umbrella term that includes task-based measures of executive function or the magnitude of brain activity in regions such as the dorsolateral prefrontal cortex (dlPFC), ventrolateral prefrontal cortex (vlPFC), and the ventromedial prefrontal cortex (vmPFC) (Goldin et al. 2008; McRae et al. 2010). However, another potential internal resource that has received much less consideration is functional connectivity between canonical intrinsic brain networks such as the attention control network (ACN), the salience network (SN), and the default mode network (DMN). Previous research has shown that differences in inter-network connectivity in the brain may be associated with reappraising versus passively viewing emotional scenes (Sripada et al. 2014). However, there is no research regarding the individual contributions of connections between intrinsic functional networks in emotion regulation.

On a regional level, reappraisal is generally associated with greater activation of lateral frontoparietal networks, which include aspects of the dlPFC and vlPFC (McRae et al. 2010; Ochsner et al. 2012). A recurring finding is that the dlPFC is associated with reappraisal as well as several other forms of emotion regulation including distraction (McRae et al. 2010; Kanske et al. 2011; Ochsner et al. 2012; Buhle et al. 2014; Silvers et al. 2015). Individual difference research has found greater activity in the dlPFC to be associated with greater regulation ability (Drabant et al. 2009; Greening et al. 2014; Horner et al. 2024). Regional analyses of brain activation have also implicated the vlPFC (Goldin et al. 2008; Wager et al. 2008; Buhle et al. 2014; Silvers et al. 2015) in emotion regulation. In addition, aspects of the parietal lobe are activated in some reappraisal studies (McRae et al. 2010; Kanske et al. 2011; Buhle et al. 2014). While many of the previous findings do not evaluate functional connectivity differences, they allude to the potential networks involved in emotion regulation, in particular, the frontoparietal networks and the ACN. The left frontoparietal network is believed to be involved in language and working memory performance, while the right frontoparietal network is associated with memory, including working memory, divided attention, and inhibition (Laird et al. 2011). Previous research has also shown that the frontoparietal network contributes to emotion regulation performance (Schweizer et al. 2013; Wessing et al. 2015; Moreira et al. 2021). Additionally, the ACN is associated with attentional orienting, and attention and cognitive control (Roye et al. 2020). Moreover, the ACN is implicated in reappraisal (Viviani 2013; Sripada et al. 2014).

Reappraisal is also associated with the suppression or inhibition of areas such as the insula and amygdala. Univariate analyses find decreases in insula activity during the down-regulation of emotion (Goldin et al. 2008; Min et al. 2022). The insula is commonly associated with the SN, which is associated with attention to emotional stimuli (Menon and Uddin 2010) and emotion regulation (Morawetz et al. 2016c; Denny et al. 2018). In addition, univariate activation studies show that the down-regulation of negative emotions by reappraisal is associated with the down-regulation of the amygdala (Goldin et al. 2008; McRae et al. 2010; Kanske et al. 2011; Ochsner et al. 2012; Buhle et al. 2014). The amygdala is typically associated with the limbic network, which plays a role in emotional responses (Laird et al. 2011; Lee and Telzer 2016). The limbic network also decreases in activity during emotional down-regulation (Moreira et al. 2021).

The vmPFC is another important region in either the down-regulation of the amygdala (Johnstone et al. 2007; Hermann et al. 2021) or the up-regulation of positive affect (Greening et al. 2014), especially owing to its robust and direct connections to the amygdala, unlike aspects of the lateral frontoparietal lobes (Mitchell 2011). Additionally, the posterior medial cortex (PMC) can be more active during reappraisal (McRae et al. 2010). From an intrinsic network perspective, the vmPFC and PMC are core aspects of the DMN. The DMN also includes secondary aspects such as the angular gyrus and hippocampus (Buckner et al. 2008; Laird et al. 2011). It is implicated in mind wandering (Fox et al. 2015), self-referential processing (Gusnard et al. 2001; Finn 2021), episodic memory, imagining the future (Buckner et al. 2008), and internally focussed cognition more broadly (Spreng et al. 2010). The DMN is also associated with the processing of emotion (Saarimäki et al. 2022), with weaker connectivity within the DMN associated with less emotionality during habituation (Min et al. 2024). With respect to emotion regulation, greater emotion regulation ability is associated with less positive connectivity between the PMC and the vmPFC (Fresco et al. 2017; Lieberman et al. 2023). Moreover, resting-state functional connectivity within the DMN has been associated with greater self-reported reappraisal and suppression (Chen et al. 2024). While these results may seem mixed, they suggest that the DMN plays a nuanced and underappreciated role in emotion regulation.

As these networks all seem to contribute to emotion regulation, it is possible that the pattern of connections between networks also contribute to emotion regulation. For example, task-based connectivity analyses during emotion regulation have found negative connectivity between lateral and medial prefrontal areas and the amygdala (Kanske et al. 2011) as well as the insula (Li et al. 2021), such that when activity in prefrontal areas increases, activity in the amygdala and insula decreases. Thus, the negative (i.e. anticorrelated) connectivity between the frontoparietal networks and emotion-focused networks such as the SN and the limbic network may contribute to emotion regulation ability. Previous research has also found that connections between the SN and DMN are associated with emotion regulation in the context of anxiety and trauma (Fresco et al. 2017; Lieberman et al. 2023). Moreover, frontoparietal networks and the DMN tend to have negative connectivity during externally-oriented cognitive tasks (Spreng et al. 2010). As reappraisal is a cognition-based form of emotion regulation (Gross 2002), it may serve as an internally focussed cognitive task, while passive viewing is an externally focussed perceptual task, with minimal cognitive demands. Furthermore, greater negative correlation between the ACN and DMN is associated with distractor suppression (Rosenberg et al. 2017). Together, these findings imply that there may be a reliable pattern of inter-network connectivity of the intrinsic functional networks during reappraisal versus the passive viewing of emotional scenes.

The studies discussed in the previous paragraphs provide evidence that multiple networks may be involved in emotion regulation. Intrinsic network functional connectivity analyses provide a holistic method of determining contributions of network interconnectivity that might not be seen with a univariate analysis of brain activity, or in seed-based psychophysiological interaction (PPI) connectivity analyses. One recent study has shown evidence that functional connectivity between networks differs between emotion regulation and passive viewing conditions, involving visual networks, frontoparietal networks, the ACN, and the DMN (Sripada et al. 2014) However, Sripada et al. (2014) focused on the univariate differences of functional connections (i.e. edges), rather than considering how the pattern of connections might together be related to emotion regulation.

Multivariate pattern analysis (MVPA) is an analytic approach that serves as a robust method of quantifying the contribution of multiple explanatory variables (e.g. brain regions or voxels) to a given cognitive process (Norman et al. 2006; Gabrieli et al. 2015). This approach has been helpful in the emotion regulation literature to determine brain activity patterns of emotion regulation (Morawetz et al. 2016a; Horner et al. 2024), and to differentiate between reappraisal and distraction (Martins et al. 2014). In addition, MVPA can also be used to elucidate whether the pattern of inter-network functional connectivity is predictive of distinct cognitive processes (Heinzle et al. 2012). However, to our knowledge, no research to date has evaluated whether the cognitive reappraisal of negative scenes can be distinguished from the passive viewing of negative scenes using MVPA of inter-network functional connectivity data.

The purpose of the present study was to test the hypothesis that MVPA of task-based inter-network functional connectivity between intrinsic canonical networks can be used to predict whether participants are reappraising versus passively viewing negative scenes. To address our primary hypothesis, we implemented two specific network-based analyses. First, we investigated univariate connectivity differences between canonical intrinsic networks as identified using a group independent components analysis (ICA) with a specific interest in the ACN, frontoparietal networks, SN, limbic network, and DMN. We expected that during reappraisal, greater negative connectivity (i.e. greater anticorrelation) would be observed between the emotion-focused networks (i.e. the SN and the limbic network) and the attentional and cognitive control networks (i.e. the ACN and the frontoparietal networks) compared to during passive viewing. We also expected that during reappraisal, greater positive connectivity between the ACN and DMN would be observed compared to during the passive viewing condition, due to its role in internally directed cognitive processing. Second, and most importantly, using MVPA and leave-one-subject-out cross-validation, we tested the prediction that the patterns of inter-network task-based functional connectivity could predict whether participants were reappraising versus passively viewing negative scenes. We expect that the most consistently reliable connections in this model will include the ones listed above.

## Methods

### Participants

33 participants were recruited from the community in and around York University, Toronto, CA. Of the participants, one participant was removed from the study due to an incidental finding in the anatomical scan, while one participant was removed for not completing the emotion regulation task, leaving 31 participants (21 female, nine male, one unspecified; age 18–36, M = 21.03, one participant did not specify age). The sample size was selected based on previous research that sought to identify independent components (Lee et al. 2017b), create connectivity matrices to determine differences in functional states (Rosenberg et al. 2015), and use linear support vector machine (SVM) classification (Pariyadath et al. 2014). Participants were screened to ensure that they could safely enter the MRI scanner. All participants gave informed consent, and the experiment was approved by the University of Manitoba Research Ethics Board (HE2023–0359).

### Procedure

**Fig. 1:**
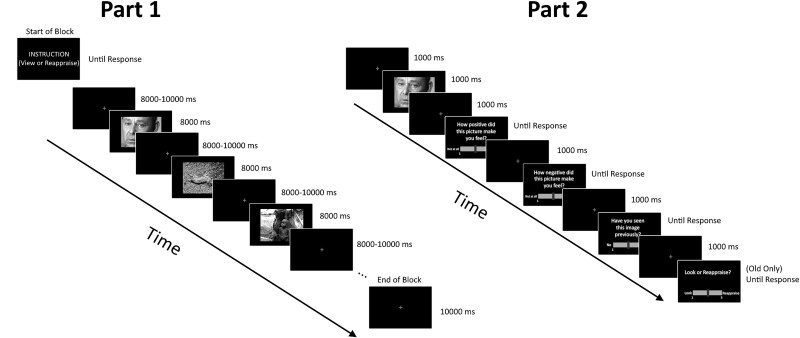
An example of a block of trials (left) from a run of the emotion regulation task in part 1, and an example trial from part 2 (right) outside the scanner. Each run in part 1 began with an instruction. Participants carried out that instruction for the entire run of trials for each of the presented images. In part 2, participants viewed images from part 1 along with new images. Participants were asked to rate how the images made them feel, whether they had seen the images before, and for old images, which instructions they followed when they saw the image in part 1.

Participants completed a modified emotion regulation task optimized to measure task-based functional connectivity using tools more commonly associated with resting-state functional MRI (fMRI) analysis, as recommended by Finn (2021). We used an exaggerated block design in which participants spent one 284-second fMRI run passively viewing negative images, followed by one 284-second run down-regulating images using reappraisal. We chose this method despite order effects because if the Reappraise run were first, participants would be more likely to implement that strategy during the View run. At the beginning of the first run, participants were given an instruction to view the images. The View instruction read as follows: “In a moment, you will see some photos on the screen. Please pay close attention to the photos. On these trials, you will simply view the images, without performing any method of regulation.” At the beginning of the second run, participants were given an instruction to reappraise the images. The Reappraise instruction read as follows: “In a moment, you will see some photos on the screen. Please pay close attention to the photos. On these trials, you will reappraise your interpretation of the images. Reappraisal is a form of emotion regulation in which you attempt to reinterpret an emotional stimulus. When instructed to reappraise, try to view the image in a way that makes it seem less negative. This may include constructing a positive outcome (a person injured soon received medical help and made a full recovery, OR this person sacrificed themselves to save other people). You may also focus on an aspect of the image that may not be as bad as it initially seemed. It is important that you do not look away from the image on the screen.” In each run, participants viewed 16 negative images. Before each image, a fixation of variable duration (i.e. jittered) between 8,000–10,000 ms appeared. Then each negative image appeared on screen for 8,000 ms, and participants carried out the instruction they were provided at the beginning of the run (i.e. View or Reappraise). Each run ended with a final 10-second fixation. See Fig. 1 for a visual depiction of an example block of trials from a run. To avoid confounding the signal with motion-related confounds, this task design did not include any trial-by-trial instructions or behavioral responses (e.g. Likert responses). However, we collected behavioral responses outside the MRI scanner, as changes in emotional responses to regulated stimuli are sustained afterwards (Erk et al. 2010).

Once outside the scanner, a behavioral task was conducted to evaluate self-reported affect and recognition memory for the previously presented images. This allowed for the evaluation of the success of reappraisal and allowed for the quantification of how well participants followed instructions to View and Reappraise. Specifically, participants completed a self-paced affect rating and recognition memory test in which they viewed all 32 of the previously seen images as well as 32 new images (see Fig. 1). On each trial, participants saw one of the images followed by a series of questions relating to the image. First, they rated how positive and how negative each image made them feel on a 1–7 Likert scale for all images. Next, recognition memory of participants was assessed by having participants first judge whether the image was old or new. Then, for all old images, participants were asked to make a source decision judgment to evaluate their degree of memory recollection. Specifically, participants indicated whether the old image was shown in the View run or the Reappraise run. Previous research finds that reappraisal effects are sustained (Erk et al. 2010; Denny et al. 2015; Hermann et al. 2021), meaning that ratings outside the scanner can be used as an indirect measure of subjective feelings while in the scanner.

### Stimuli

64 images were selected from the International Affective Picture System, the Nencki Affective Picture System, and the Geneva Affective Picture Database datasets (Lang et al. 1997; Dan-Glauser and Scherer 2011; Marchewka et al. 2014). Of the 64 images, 32 were high intensity, half of which had high affordances for reappraisal, and the other half had low affordances for reappraisal. The remaining 32 images were low intensity, half of which had high affordances for reappraisal, and the other half had low affordances for reappraisal. Images were randomly assigned to the view, reappraise, and new conditions, with 16 images being in each of the View and Reappraise conditions, and 32 images being in the new condition. We selected the images from a range of affordances and intensity to show effects in a wide range of negative emotionality and emotion regulation performance (Suri et al. 2018; Horner et al. 2023). Images were randomly assigned to matched sets so that an equal number of images of each of the four types was in each group.

### Skin conductance response

As we did not have participants complete Likert ratings in the MRI scanner, we opted to measure skin conductance response (SCR), an indirect measure of emotional arousal when looking at emotional images (Lang et al. 1998; Wood et al. 2014) that does not confound our blood-oxygen-level-dependent (BOLD) signal recordings. Similar to previous research in the lab combining concurrent psychophysiology and fMRI (Greening et al. 2022; Burleigh and Greening 2023), electrodermal activity was recorded with the Biopac MP-150 system and AcqKnowledge software (BIOPAC systems, Goleta, CA, USA) and was sampled at 1000 Hz. Two Ag/AgCl laminated, carbon composition contact electrodes with a conductive saline-based gel (BIOPAC GEL101) were placed on the fingertips of the fourth and fifth fingers of the non-dominant hand. Analyses of SCR signals were carried out in MATLAB R2018a (Version 9.4). A 0.15 Hz low-pass Butterworth filter was used to remove high-frequency radiofrequency noise from the scanner (Nagai et al. 2004). Next, a 0.01 Hz high-pass Butterworth filter was used to remove the slow-wave drift (Bach et al. 2010). The time series were then down-sampled to 10 Hz. Trial-wise baseline detrending was conducted by subtracting the baseline (mean electrodermal activity recorded one second prior to image onset) from the rest of the trial segment. Next, SCRs were calculated through a trough-to-peak analysis strategy. Specifically, a minimum SCR value was identified in the first second after image onset. The maximum SCR value was identified between the 1–8 s time window after stimulus onset. An SCR that did not cross a 0.02 μS threshold was set to zero (Boucsein et al. 2012). To increase normality, the difference scores were then square root transformed (Burleigh et al. 2022). SCR data from two participants was excluded as they were non-responders, in that there were no detectable SCRs to any trial. Moreover, the inclusion of these two participants in the analysis does not change the inferential findings.

### Brain imaging acquisition

The experiment was completed at the York MRI Facility using a 3 T Siemens PrismaFit scanner with a 32-channel head coil. A T1-weighted magnetization-prepared rapid gradient-echo whole brain sequence was used to acquire anatomical images (repetition time: 2300 ms, echo time: 2.26 ms, voxel size 1x1x1-mm, slices, 192, flip angle 8^°^). Functional images were acquired across two runs using a T2*-weighted multi-echo planar imaging sequence for each run (repetition time: 1000 ms, echo times: 12 ms, 30 ms, and 48 ms, voxel size 3 × 3 × 3 mm, 52 slices, flip angle 50°, multi-band acceleration factor: 4).

### Analysis

For each participant’s emotion regulation runs, fMRIPrep (version 1.1.22) was performed for preprocessing (See Supplemental for a detailed boilerplate). In summary, our anatomical brain data was skull-stripped, segmented, and normalized to standard space. For functional data, we estimated head-motion parameters (i.e. transformation matrices, and six corresponding rotation and translation parameters) (FSL, Jenkinson et al. 2002), co-registered data using boundary based regression and six degrees of freedom (Greve and Fischl 2009), flagged any motion outliers with framewise displacement of 0.9 mm or more, and extracted physiological regressors for component-based noise correction (CompCor, Behzadi et al. 2007).

#### Univariate whole brain activation analysis

For our univariate activation analysis, we performed some additional preprocessing and analyses using FEAT (FMRI Expert Analysis Tool) Version 6.00, part of FSL (FMRIB’s Software Library, www.fmrib.ox.ac.uk/fsl). The analyses in this section were only used to determine univariate effects and were not used for the network connectivity analyses. Spatial smoothing was carried out using a Gaussian kernel of 6.0 mm full width at half maximum (FWHM), high-pass temporal filtering (Gaussian-weighted least-squares straight line fitting, with sigma = 100.0 s), and time-series statistical analysis were carried out using FILM with local autocorrelation correction (Woolrich et al. 2001).

For each individual participant’s level 1 analysis, each run was modeled separately. A double-gamma hemodynamic response function convolution was used for the 4 explanatory variables of image type (i.e. high intensity + high affordances, high intensity + low affordances, low intensity + high affordances, low intensity + low affordances) inputted in Custom (3 column format) basic shape. Temporal derivatives of each explanatory variable were also included. Nuisance variables were also included in each level 1 for the purpose of denoising. Specifically, we included the first five components of aCompCor (Muschelli et al. 2014), the cosine, the three translations and three rotations, and all motion outliers using an outlier detection of 0.9 mm (Siegel et al. 2014) as regressors of no interest. Finally, for each run (i.e. the View run, and the Reappraise run), we compute the key contrast such that each of the four explanatory variables of image type were combined with equal weighting and contrasted with 0 (i.e. baseline).

We then performed a standard univariate activation analysis using FLAME stage 1 (Beckmann et al. 2003; Woolrich et al. 2004; Woolrich 2008). Z (Gaussianised T/F) statistic images were thresholded using clusters determined by Z > 3.1 and a (corrected) cluster significance threshold of *P* = 0.05 (Worsley 2001). The whole-brain univariate analysis was used to determine the Reappraise > View contrast and the View > Reappraise contrast. For completed reporting and details of the whole-brain univariate results, the uncorrected whole-brain univariate maps can be found at https://neurovault.org/collections/VFVNUBCU.

#### Network connectivity analyses

**Fig. 2:**
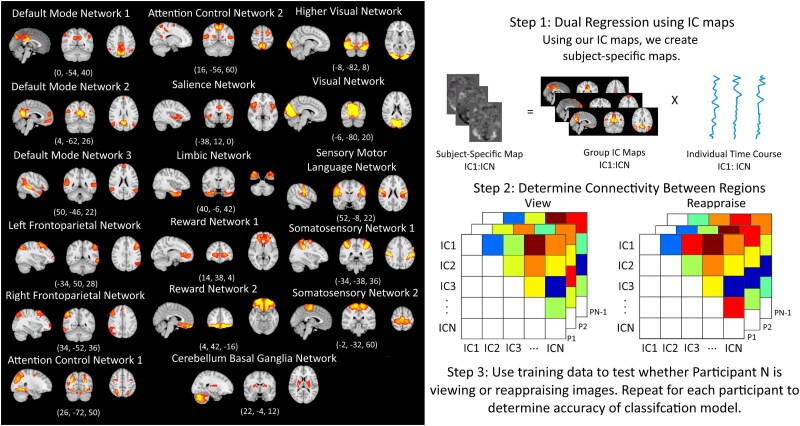
Left: Canonical networks derived from our ICA with z thresholds of 5 < z < 10. Right: Steps to how we completed our connectivity analysis.

The present study focused on task-related functional connectivity (Finn 2021). The fMRI connectivity analysis was completed using a method previously used by our lab (Roye et al. 2020). For the network fMRI connectivity analysis, we used the optimally weighted data preprocessed by fMRIPrep. We smoothed the filtered data using a Gaussian kernel of 6.0 mm FWHM. We corrected for motion using reg_filt by regressing out the first five components of aCompCor (Muschelli et al. 2014), the cosine, the three translations and three rotations, and all motion outliers using an outlier detection of 0.9 mm. Then, we concatenated the participant-denoised data into a 4D file and used MELODIC-ICA to perform a group-wise ICA for 25 independent components (ICs). We chose 25 as previous research has found 18–23 ICs corresponding to functional brain networks (Beckmann et al. 2005; Laird et al. 2011; Lee et al. 2017b) can be identified using this approach. We wanted to ensure we could identify the canonical resting-state brain networks while also accounting for the likelihood of several ICs corresponding to artifactual information that would need to be discarded. Further, we opted to use ICA instead of a multi-seed-based PPI analyses to drastically decrease the number of connections (a.k.a., edges) tested (Laird et al. 2011; Lee et al. 2017b; Roye et al. 2020). Next, we identified canonical networks by comparing our MELODIC file to intrinsic connectivity networks from previous research (Laird et al. 2011), in which we used fslcc to select 17 ICs that correlated with the intrinsic connectivity networks with an r-value of 0.3 or greater. Network names were determined based on established networks associated with ICNs in previous research (Laird et al. 2011; Lee et al. 2017b; Roye et al. 2020). Networks selected were three DMN subnetworks (DMN1, DMN2, and DMN3), the left and right frontoparietal network (Left FP and Right FP), two attention control networks (ACN1 and ACN2), the SN, the limbic network (LIMB), two reward networks (REW1 and REW2), the cerebellum basal ganglia network (CBG), the higher visual network (HVIS), the visual network (VIS), the sensorimotor language network (SML) and two somatosensory networks (SSN1 and SSN2) (See Fig. 2 for selected networks). To provide the complete details regarding each network map, we provide the unthresholded whole-brain maps of each of the selected ICs, including r-values of the cross-correlations, at https://neurovault.org/collections/VFVNUBCU. Additionally, r-values of the cross-correlations for each network can be found in the supplemental materials.

Next, we used dual regression (Beckmann et al. 2009; Nickerson et al. 2017) to generate subject-specific versions of the spatial maps, and associated timeseries based on the results of the ICA (See Fig. 2). For each subject, the network IC maps developed using MELODIC ICA are regressed into their 4D spatial–temporal maps, where the pseudoinverse of the spatial map matrix is multiplied by the spatial–temporal maps (Nickerson et al. 2017). This results in a series of subject-specific timeseries, one for each network, which show activity during the time course at the relevant voxels. The pseudoinverses of these timeseries are then regressed into the same 4D spatial map (Nickerson et al. 2017), resulting in a subject-specific spatial map for each network.

We next used FSLNets (https://fsl.fmrib.ox.ac.uk/fsl/docs/#/resting_state/fslnets) to create an unregularized partial correlation matrix for the Reappraise and View conditions across each subject (Smith et al. 2013). We opted to use partial correlation instead of full correlations because partial correlations show an approximation of the direct connections between networks (Smith et al. 2011, 2013). Specifically, variance normalization was performed on each separate timeseries for each participant. Next, the timeseries of each artifactual nodes (i.e. ICs) was regressed out of the timeseries of each node we retained as our canonical networks using the aggressive cleanup option. The timeseries of each artifactual nodes was then discarded. Next, an unregularized partial correlation was computed for each pair of nodes then normalized using Fisher’s R-to-Z transformation. This produced a connectivity matrix for each participant with z-values representing the strength of connectivity between pairs of nodes (i.e. our canonical networks).

Next, we used scikit-learn (Pedregosa et al. 2011) to conduct a connectivity-based MVPA to determine if we could predict whether a participant is passively viewing or reappraising an image based on their flattened connectivity matrix (Ullman et al. 2014). We used a leave-one-subject-out cross-validated linear support vector machine (SVM) classification model, C = 1.0, (Pariyadath et al. 2014). Connectivity values for the model were derived from Fisher z-values of the partial correlation. To determine if our classification accuracy was significantly greater than chance, 5000 permutations were run randomizing the reappraise and view labels.

In an additional analysis, we also performed 10% feature selection by extracting the top 10% of the connections with the greatest linear coefficients across each fold (Pariyadath et al. 2014), with 14 connections being chosen for each fold. The odds of being selected for each fold would be 10.29%, and edges that appeared in the top edges at least 11 times were considered consistently meaningful linear coefficients, as they appeared in the top consistently more often than chance (*P* < 0.000005).

## Results

### Behavioral results

#### Self-reported ratings of affect

**Fig. 3:**
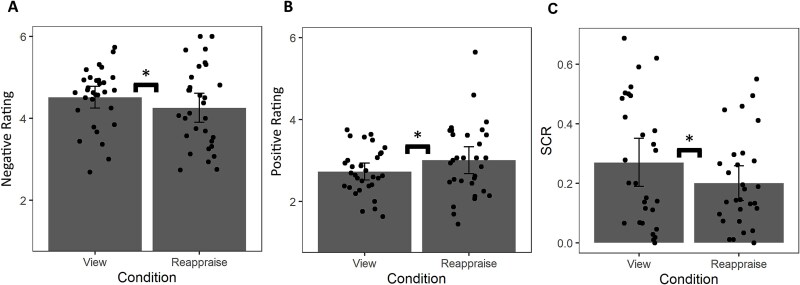
Differences in A) negative rating, B) positive rating, and C) SCR between reappraise and view conditions. Error bars represent 95% confidence intervals. Dots represent the response of individual participants. An asterisk (*) indicates a *p*-value less than 0.05.

Using a paired samples t-test, we found a significant effect of instruction on negative ratings, such that images that were passively viewed (M = 4.516, SD = 0.724) were rated as more negative than images that were reappraised (M = 4.258, SD = 0.974), t(30) = 2.252, *P* = 0.032, d’ = 0.301 (See Fig. 3A). We also found a significant effect of instruction on positive ratings, such that images that were passively viewed (M = 2.726, SD = 0.567) were rated as less positive than images that were reappraised (M = 3.006, SD = 0.892), t(30) = −2.221, *P* = 0.034, d’ = 0.374 (See Fig. 3B).

#### Memory measures

Overall accuracy on the memory task was 94.10%. A paired samples t-test showed no significant differences in accuracy between old images (M = 95.16%, SD = 7.07%) and new images (M = 93.04%, SD = 10.70%), t(30) = 1.079, *P* = 0.289, d’ = 0.233. We found a significant effect of instruction on memory, such that images that were passively viewed (M = 93.35%, SD = 9.54%) were remembered with worse accuracy than images that were reappraised (M = 96.98%, SD = 5.56%), t(30) = −3.0571, *P* = 0.0047, d’ = 0.465. Overall source decision accuracy was 77.79%, and a paired samples t-test found no significant differences in source decisions between passive viewing (M = 79.18%, SD = 23.69%) and reappraisal (M = 76.46%, SD = 28.47%), t(30) = 0.2976, *P* = 0.7681, d’ = 0.104.

### Skin conductance results

Using a paired samples t-test, we found a significant reduction of SCR to the negative images when reappraising (M = 0.20, SD = 0.15) compared to passive viewing of the images (M = 0.27, SD = 0.21), t(28) = 2.24, *P* = 0.034, d’ = 0.415 (See Fig. 3C).

### Univariate analysis results

**Fig. 4:**
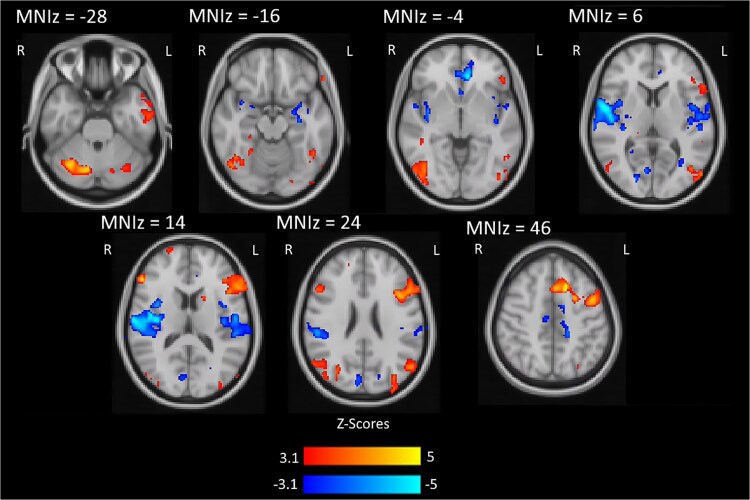
Blood-oxygen-level-dependent response for the whole brain analysis of differences between reappraise and view trials. Red-yellow clusters show reappraise > view activation while blue clusters show view > reappraise activation. Significant activation clusters are displayed on MNI 2 mm brain images and are the result of the group-level whole-brain analysis conducted with a cluster-forming threshold of z > 3.1 and a (corrected) cluster size probability of *P* < 0.05.

***Activation Results:*** See Fig. 4 and Table 1 for the full univariate activation results. Notably, we found significantly greater activity for Reappraise compared to View in bilateral aspects of the dlPFC, bilateral aspects of the occipital cortex, the left dlPFC, and right aspects of the cerebellum. Conversely, we observed significant reductions in activation during Reappraise versus View in aspects of the bilateral hippocampus, amygdala, and insula.

**Table 1:** Significantly active clusters of differential BOLD signal contrast between reappraise and view conditions. Clusters organized by size. Voxels represents the number of contiguous (adjacent faces) voxels in a cluster. Z-MAX is the maximum Z-value of that cluster, and the coordinates (MNI space) are the location of the voxel with Z-MAX in the cluster. The significantly activity clusters are the results of the group-level whole-brain analysis conducted with a cluster-forming threshold of z > 3.1 and a (corrected) cluster size probability of *P* < 0.05.

Cluster Index	Voxels	Location	Z-MAX	Z-MAX X (mm)	Z-MAX Y (mm)	Z-MAX Z (mm)
Reappraise > View						
1	4630	L Superior Frontal Gyrus/L Middle Frontal Gyrus/L Inferior Frontal Gyrus (dlPFC/vlPFC)	5.05	−14	18	46
2	1373	R Cerebellum/R Lateral Occipital Cortex (inferior)	4.79	34	−64	−32
3	686	R Lateral Occipital Cortex (superior)	4.86	26	−72	32
4	522	L Lateral Occipital Cortex (superior)	4.32	−36	−76	32
5	314	L Lateral Occipital Cortex (inferior)	4.05	−54	−78	6
6	264	R Inferior Frontal Gyrus (vlPFC)	4.82	54	32	14
7	201	L Lateral Occipital Cortex (superior)	4.21	−54	−68	24
8	188	L Middle Temporal Gyrus (anterior)	4.94	−50	−8	−22
9	169	L Cerebellum	3.93	−28	−68	−34
View > Reappraise						
1	2769	R Central Operculum Cortex/R Parietal Opercular Cortex/R Insula/R Supramarginal Gyrus/R Precentral Gyrus	5.47	56	0	4
2	1527	L Central Operculum Cortex/L Parietal Opercular Cortex/L Insula/L Supramarginal Gyrus/L Amygdala	4.63	−72	−24	28
3	299	L Paracingulate Gyrus/L Anterior Cingulate Gyrus	5.15	−8	36	−6
4	186	R Intracalcine Cortex/R Cuneal Cortex	3.98	8	−72	6

### Multivariate brain imaging results

**Fig. 5:**
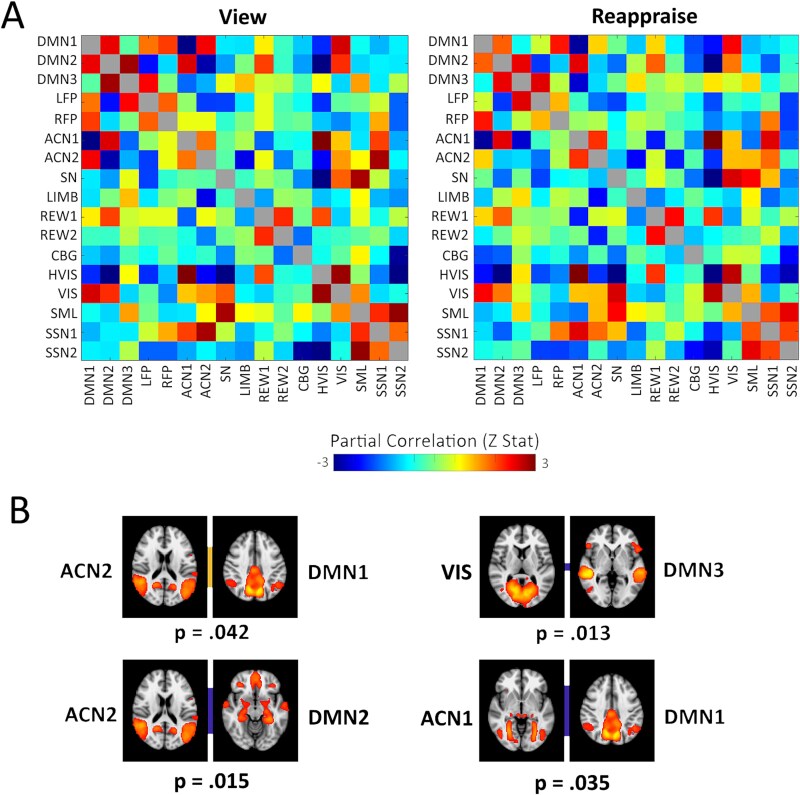
A) Z-stats of partial correlations between canonical networks in the view (left) and reappraise (right) conditions and B) most significant edges in the linear model for view-reappraise. The orange line means that the correlation between the networks was reduced in the reappraise condition. The blue line means that the anticorrelation between the networks was reduced in the reappraise condition.

**Fig. 6:**
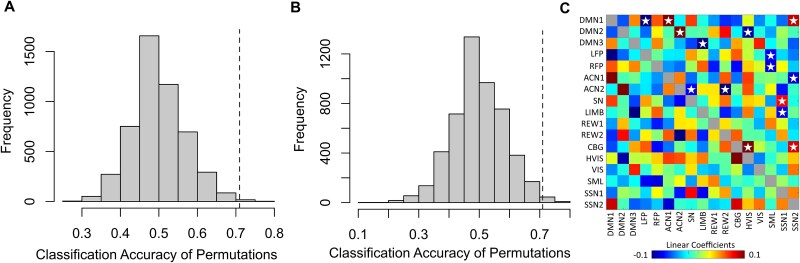
A, B) histogram comparing our observed classification accuracy (dashed line) to the null distribution of accuracies produced from the permutation test using all edges (A), and most consistently meaningful edges (B). C) Linear coefficients of the support vector classification of the 17 canonical networks. Positive linear coefficients are associated with the prediction of reappraisal resulting from more positive z-values during the reappraisal condition, including when the inter-network connections are less negative during reappraisal compared to view. Negative linear coefficients are associated with the prediction of view resulting from more positive z-values during the view condition, including when the inter-network connections are less negative during view compared to reappraisal. In the upper triangle, the most reliable features are labeled with a white star.

Fisher transformed z-values of partial correlations for Reappraise and View conditions can be seen in Fig. 5A. A 5000-permutation support vector classification with leave-one-subject out cross-validation found that connectivity between the 17 canonical brain networks predicted whether a participant was reappraising or passively viewing an image better than chance, accuracy = 70.97%, *P* = 0.0040 (Fig. 6A). When 10% feature selection was performed with the most consistently meaningful linear coefficients, prediction accuracy improved, accuracy = 79.03%, *P* = 0.0004.

#### Functional connectivity results

A cross-subject general linear model was performed within subjects with contrasts Reappraise > View and View > Reappraise and correction for multiple comparisons was done using FDR correction across the 136 connections, and corrected p-values were determined using 5000 permutations. DMN2 and ACN2 were more anticorrelated in the View condition than the Reappraise condition p_corr_ = 0.0150. DMN1 and ACN1 were more anticorrelated in the View condition than the Reappraise condition p_corr_ = 0.0354. DMN3 and the visual network were more anticorrelated in the View condition than the Reappraise condition p_corr_ = 0.0128. DMN1 and ACN2 had greater correlation in the View condition than the Reappraise condition p_corr_ = 0.042 (see Fig. 5B).

Linear coefficients can be seen in Fig. 6C, with the extracted edges that contribute most to the model being shown in Table 2. Edges positively predictive of reappraisal were those connections that resulted in greater positive weights, either due to greater correlation or less anticorrelation in the Reappraise condition compared to View. Of note, we found lesser inter-network anticorrelations between DMN1 and ACN1, as well as DMN2 and ACN2 in the Reappraise condition compared to the View condition. Likewise, edges negatively predictive of reappraisal entailed connections that resulted in greater negative weights, either due to lesser inter-network correlation or greater inter-network anticorrelation in reappraisal versus View. Notably, we observed greater inter-network anticorrelation between ACN2 and SN, as well as greater inter-network anticorrelation between ACN2 and REW2 in the Reappraise condition compared to the View condition.

**Table 2:** Functional connectivity edges that made a consistently meaningful contribution to the full model predicting reappraise vs view.

	Edge	Linear Coefficient	Mean Z (View)	Mean Z (Reappraisal)
***Edges positively predictive of reappraisal***				
Greater inter-network correlations in Reappraisal vs. View				
	SN + SSN1	0.075	0.364	1.118
Lesser inter-network anticorrelation in Reappraisal vs. View				
	DMN2 + ACN2	0.126	−2.751	−1.067
	DMN1 + ACN1	0.114	−4.098	−2.857
	CBG + HVIS	0.113	−0.667	−0.359
	DMN1 + SSN2	0.086	−0.835	−0.647
	CBG + SSN2	0.082	−2.867	−2.236
				
***Edges negatively predictive of reappraisal***				
Lesser inter-network correlation in Reappraisal vs. View				
	DMN3 + LIMB	−0.109	1.114	0.928
	DMN1 + Left FP	−0.104	1.555	0.424
Greater inter-network anticorrelation in Reappraisal vs. View				
	ACN2 + REW2	−0.135	−1.490	−2.106
	DMN2 + HVIS	−0.092	−3.220	−3.558
	ACN1 + SSN2	−0.085	−1.253	−1.713
	RFP + SML	−0.082	−0.958	−1.360
	LIMB+SSN1	−0.078	−1.191	−1.623
	Left FP + SML	−0.077	−0.087	−0.620
	ACN2 + SN	−0.076	−0.253	−1.108

## Discussion

The purpose of this study was to test the general hypothesis that differences in inter-network connectivity are predictive of the reappraisal versus passive viewing of negative scenes. We expected inter-network connectivity differences between emotional networks (i.e. SN and limbic network), cognitive networks (i.e. ACN and frontoparietal networks), and the DMN. Broadly, our findings support this general hypothesis. First, using a cross-section general linear model, we observed differences in connectivity between aspects of the DMN and aspects of the ACN, as well as between aspects of the DMN and the visual network, during passive viewing versus reappraisal, showing partial support for our hypothesis that reappraisal would be associated with greater positive connectivity between the ACN and DMN. Second, we demonstrated that we could predict whether someone was reappraising or passively viewing an image based on the pattern of inter-network connectivity, as was hypothesized. We found that a multivariate pattern analysis of inter-network connections between 17 canonical networks (136 edges) using support vector classification predicted whether one was reappraising or viewing a negative image greater than chance, with connections involving frontoparietal networks, ACN, SN, limbic network, and the DMN, as well as several other networks contributing to the model.

The behavioral task and SCR results confirmed the effectiveness of the reappraisal manipulation. We found that images were rated as more negative and less positive in the View condition compared to the reappraisal condition, which is consistent with much of the previous literature. Moreover, this suggests that our use of a longer exaggerated block design in which participants receive the regulation instruction only once at the start of the block of trials for a given run did not meaningfully affect the emotion regulation task. Our findings are also consistent with previous research showing that reappraisal is effective and that the effects of regulation are sustained over time (Erk et al. 2010; Denny et al. 2015; Hermann et al. 2021). The behavioral results also revealed that memory for reappraised images was greater than memory for passively viewed images, which is consistent with previous literature (Dillon et al. 2007; Wang et al. 2017; Yeh et al. 2020). One possible explanation for this is that reappraisal requires greater attentional control compared to passive viewing, which in turn could lead to improved memory encoding and later retrieval of the reappraised emotional information (Dillon et al. 2007). There were no significant differences in source monitoring accuracy between the View condition and the Reappraise condition, suggesting that participants remembered the conditions in which they saw the images equally across the two conditions. There was, however, also a significant reduction in SCR when reappraising versus viewing the negative stimuli. This is consistent with emotion regulation successfully down-regulating SCR activity in studies involved fear-related stimuli (Delgado et al. 2008; Greening et al. 2022) and is consistent with previous observations that reappraisal can reduce the magnitude of SCRs to negative images (Driscoll et al. 2009; Urry et al. 2009). Additionally, behavioral ratings and brain activity were indeed affected by our emotion regulation paradigm, suggesting that our emotion regulation paradigm was effective.

Consistent with meta-analyses of reappraisal and emotion regulation (Buhle et al. 2014), the univariate activation analysis revealed increased activation in bilateral frontoparietal regions including dlPFC and vlPFC, and decreased activation in the amygdala, insula, vmPFC and hippocampus. These findings confirmed that our unique emotion regulation task was consistent with previous emotion regulation tasks and that areas associated with various intrinsic networks are differentially recruited during emotion regulation by reappraisal versus passive viewing.

Our network results revealed several inter-network connections that were negative predictors of reappraisal. In other words, connections in which greater anticorrelations, or lesser positive correlations, were observed during reappraisal versus passive viewing of negative images. Importantly, these results provided partial support for our first specific prediction that the ACN and frontoparietal (FP) networks would be more anticorrelated with the SN and limbic networks during reappraisal versus passive viewing of negative images. Specifically, the MVPA analysis demonstrated that the edge between the ACN2 and SN was a reliable negative predictor of reappraisal (i.e. a positive predictor of View), in which there was a larger anticorrelation between these networks during reappraisal compared to View, partially supporting our hypothesis. The ACN2 in the present study included frontoparietal areas previously associated with emotion regulation such as the bilateral dlPFC and temporoparietal junction (Buhle et al. 2014). Moreover, the anticorrelation of the SN with ACN2 in the present study is consistent with previous findings of reduced anterior insula activity during reappraisal of negative stimuli (Goldin et al. 2008). From a network perspective, the present findings involving ACN2 are consistent with previous descriptions of the ACN being associated with attention selection and inhibition (Laird et al. 2011), both of which can decrease emotional responses when necessary (Tabibnia et al. 2011; Schmeichel and Tang 2015).

However, inconsistent with our predictions, we found no evidence of greater anticorrelations or lesser positive correlations during reappraisal between the FP networks and either the SN or limbic network, nor between the ACN and the limbic network. Regarding the lack of findings between the frontoparietal networks and both the SN and limbic network, there are at least two potential considerations. First, the connections between emotion-focused networks (i.e. the limbic network and SN) and the frontoparietal network may be indirect, working through other networks. This may likewise explain the lack of significant differences in connectivity between the ACN and limbic network. Second, and more broadly for the inter-network analyses, our study used intrinsic networks and allowed for relatively fewer total pairwise comparisons compared to using a large number of seeds for each network. For example, while our study had 136 edges between 17 networks derived from independent components, Sripada et al. (2014) derived several 100,000 edges using seeds from 837 ROIs. Separating out the subregions of each network may show more nuanced connectivity between intra-network in addition to inter-network regions. However, it would come at the cost of more edges to consider, which can result in the need for robust multiple comparisons correcting in univariate analyses (García 2003) and overfitting in multivariate analyses in which the number of dimensions, or explanatory variables, is far greater than the number of samples (Bishop and Nasrabadi 2006; Kriegeskorte et al. 2009). Furthermore, deriving networks using an ICA method allows for the unsupervised clustering of correlated regions irrespective of their spatial position (Beckmann and Smith 2004), and has been used in previous research for network analyses (Reineberg et al. 2015; Lee and Telzer 2016; Lee et al. 2017b, 2017a; Roye et al. 2020).

The MVPA of inter-network connectivity also revealed some unpredicted, but still interesting network relationships which were reliable negative predictors of reappraisal. Notably, the connection between ACN2 and REW2 had a greater anticorrelation during reappraisal compared to View. REW2 contained aspects of the ventral orbital frontal cortex (vOFC), which is sensitive to both negative valence (Chikazoe et al. 2014) and punishment (O’Doherty et al. 2001; Remijnse et al. 2005), rather than simply rewards. Thus, it is possible that during reappraisal, the ACN inhibits activity in the reward network, resulting in a greater anticorrelation. In addition, the connection between DMN3 and the limbic network had a reduced correlation during reappraisal. One possible explanation for this finding is that some regions within our DMN3, including lateral temporal lobe and temporoparietal junction, are also associated with theory of mind and perspective-taking (Buckner and Carroll 2007; Spreng and Grady 2009). As the limbic network is associated with emotional responses (Laird et al. 2011; Lee and Telzer 2016), it may be that when exposed to emotional images, participants are considering the emotional viewpoint displayed in the image more so in the View compared to Reappraise condition. The difference being that with reappraisal, people reorient their perspective to decrease emotional responding, resulting in lesser connectivity between these two networks during the reappraisal condition.

Related to our second prediction, the present study also found inter-network connections that were positive predictors of reappraisal versus passive viewing of negative images. These were connections in which greater positive correlations, or lesser anticorrelations, were observed. Notably, the results were consistent with our prediction that a more positive correlation would be observed between the ACN and DMN during reappraisal versus passive viewing. Specifically, both the general linear model and the MVPA analysis found that both the edge between DMN1 and ACN1 and the edge between DMN2 and ACN2 had a greater anticorrelation during View compared to reappraisal. Connectivity between the ACN and the DMN has been shown previously to increase during reappraisal (Sripada et al. 2014), similar to our findings of reduced anticorrelation during reappraisal. More generally, the pattern of connectivity we observed between the DMN and ACN, particularly in the View condition, was the typical anticorrelated connectivity reported in the literature (Menon and Uddin 2010), especially during externally focused attention tasks (Kelly et al. 2008; Spreng et al. 2010). Moreover, the relative reduction in this anticorrelation between the ACN and DMN during reappraisal was consistent with the results of Sripada et al. (2014). Regarding the reduced anticorrelations between the DMN and ACN during reappraisal, one potential explanation is that reappraisal involves a relative reduction in externally directed processing, along with a commensurate redirection of attention inwards (Buckner et al. 2008). Additionally, both the ACN1 and ACN2 of the present study included aspects of the dlPFC and of the lateral parietal lobes, which are commonly discussed in activations studies of emotion regulation (McRae et al. 2010; Kanske et al. 2011; Ochsner et al. 2012; Buhle et al. 2014; Silvers et al. 2015). On the other hand, many of the classic activation studies of emotional reappraisal do not emphasize the potential involvement of the DMN. Nevertheless, several studies have observed greater activity in the PMC during emotion regulation compared to passive viewing of emotional materials (McRae et al. 2010; Berboth et al. 2021), which is a prominent region in both the DMN1 and DMN2 in the current study. Taken together, the network perspective presented here brings new insights into a potential role of the DMN in emotion regulation that has previously been under-emphasized.

Another noteworthy finding was that compared to passively viewing negative images, reappraising images was associated with reduced negative connectivity between DMN3 and the visual network in the general linear model analysis. This supports previous research, which has shown that greater positive connectivity between the DMN and visual network was associated with reappraisal (Sripada et al. 2014). It might be the case that because the regions implicated in our DMN3 are those associated with theory of mind, and that looking at an image during reappraisal may involve reorienting one’s perspective such that the image can be viewed as less negative (Buckner and Carroll 2007; Sripada et al. 2014). Thus, rather than the DMN inhibiting the visual network, during reappraisal aspects of the DMN may be integrating some information in the visual network to help facilitate a shift in perspective.

A general observation regarding our two network analysis strategies is that the univariate connectivity analysis revealed fewer significant or reliable edges (i.e. inter-network connections) than the multivariate analysis. One possible explanation for this is that we looked for significant differences in connectivity between 136 edges and correction for that many comparisons obscured noteworthy effects. Thus, small differences in connectivity that can also contribute to regulation may have gone unseen using the univariate connectivity method. However, by using a multivariate classifier, all pair-wise combinations of network connections can provide contributions to a single model, allowing us to consider small changes in the strength of connectivity between any given two networks which may play a role in dissociating reappraisal from passive viewing (Heinzle et al. 2012). While we performed feature selection with our most reliable connections, it is worth noting that even in the analysis with all connections, the combined influence of all networks contributed to the significant full model (Greening and Mitchell 2015).

While the present study found that patterns of inter-network connectivity differed between reappraisal and passive viewing, it cannot determine the directional of causality between the networks. However, there are alternate methods we can use in future research to evaluate the potential causality of network activation. For example, dynamic causal modeling (DCM) can determine which networks are most likely activating or inhibiting other networks (Rozovsky et al. 2025), and what regions within those networks are acting on regions in other networks (Morawetz et al. 2016b; Zhang et al. 2018). Alternatively, transcranial magnetic stimulation (TMS) can be used to directly activate or inhibit networks in to evaluate causal claims regarding changes in behavior (Thakral et al. 2020; Herz et al. 2022) and brain activity (He et al. 2023). Indeed, future research could combine TMS and fMRI methods (Bestmann et al. 2008) to determine if exciting or inhibiting a given node (i.e. region) in a canonical network leads to changes in the functional connectivity between the stimulated network and other networks associated with emotion regulation.

Taken together, the present study determined that, in accordance with the SOC-ER model (Urry and Gross 2010; Opitz et al. 2012), inter-network connectivity serves as an internal factor that affects emotion regulation. The present study is the first to our knowledge to use MVPA with cross-validation to evaluate whether the broad-scale pattern of intrinsic network functional connectivity is associated with emotion regulation. Moreover, we found that inter-network connectivity can be used to predict whether someone is passively viewing or reappraising an image, which supports the idea that a distinct pattern of functional connectivity between the intrinsic networks is indicative of reappraisal. In addition, networks such as the DMN, emotional networks, and networks associated with executive function all played a role in reliably distinguishing between states of reappraisal versus passive viewing of negative scenes. Thus, emotion regulation may have a more holistic neural substrate in the brain than once thought.

## Author contributions

Scarlett Horner (Conceptualization, Data curation, Formal analysis, Investigation, Methodology, Project administration, Software, Validation, Visualization, Writing—original draft, Writing—review & editing), Thomas Rawliuk (Data curation, Formal analysis, Investigation, Project administration, Writing—review & editing, Methodology), Ryan M Ferstl (Methodology, Software, Writing—review & editing, Project Administration), Andrew L Lyons (Data curation, Investigation, Writing—review & editing, Project Administration, Funding Acquisition), Janeen Martin (Project administration, Writing—review & editing), Diana J Gorbet (Methodology, Resources, Software, Writing—review & editing), W Dale Stevens (Methodology, Resources, Writing—review & editing), and Steven Greening (Conceptualization, Funding acquisition, Methodology, Project administration, Resources, Software, Supervision, Writing—original draft, Writing—review & editing).

## Supplementary Material

Supplemental_ER_Connect_v4_bhaf161
