# Supplementary material for: It’s all connected! Multivariate pattern analysis of inter-network connectivity distinguishes between reappraisal and passive viewing of emotional scenes
Source: Cereb Cortex. 2025 Jun 25;35(6):bhaf161. doi: 10.1093/cercor/bhaf161 (PMC12192433; doi:10.1093/cercor/bhaf161)
Supplement: Supplemental_ER_Connect_v4_bhaf161 [file supplemental_er_connect_v4_bhaf161.docx]

**SUPPLEMENTARY MATERIALS**

**It’s all connected! Multivariate pattern analysis of inter-network connectivity distinguishes between reappraisal and passive viewing of emotional scenes**

Scarlett Horner^1^, Thomas Rawliuk^1^, Ryan M. Ferstl^1^, Andrew L. Lyons^1^, Janeen Martin^1^, Diana J. Gorbet^2^, W. Dale Stevens^2,3^, Steven G. Greening^1,4,*^

^1^ Brain & Cognitive Sciences, Dept. of Psychology, University of Manitoba, Canada

^2^York MRI Facility, York University, Toronto, Ontario, Canada

^3^ Department of Psychology, York University, Toronto, Canada

^4^ Centre on Aging, University of Manitoba, Canada

*Corresponding Author: Steven G. Greening, steven.greening@umanitoba.ca, Brain and Cognitive Sciences, Department of Psychology, University of Manitoba, P511 Duff Roblin, Winnipeg, MB, CA R3T 2N2; Tel.: +1 204 747 8259

What follows is boilerplate from fMRIprep, distributed under the CC0 license:

Results included in this manuscript come from preprocessing performed using fMRIPrep 23.2.2 (Esteban et al., 2018, 2019; RRID:SCR_016216), which is based on Nipype 1.8.6 (Gorgolewski et al., 2011; 2018; RRID:SCR_002502)

***Anatomical data preprocessing***

A total of 1 T1-weighted (T1w) images were found within the input BIDS dataset. The T1w image was corrected for intensity non-uniformity (INU) with N4BiasFieldCorrection (Tustison et al., 2010), distributed with ANTs 2.5.0 (Avants et al., 2008; RRID:SCR_004757), and used as T1w-reference throughout the workflow. The T1w-reference was then skull-stripped with a Nipype implementation of the antsBrainExtraction.sh workflow (from ANTs), using OASIS30ANTs as target template. Brain tissue segmentation of cerebrospinal fluid (CSF), white-matter (WM) and gray-matter (GM) was performed on the brain-extracted T1w using fast (FSL (version unknown), RRID:SCR_002823, Zhang et al., 2001). Brain surfaces were reconstructed using recon-all (FreeSurfer 7.3.2, RRID:SCR_001847, Dale et al., 1999), and the brain mask estimated previously was refined with a custom variation of the method to reconcile ANTs-derived and FreeSurfer-derived segmentations of the cortical gray-matter of Mindboggle (RRID:SCR_002438, Klein et al., 2017). Volume-based spatial normalization to one standard space (MNI152NLin2009cAsym) was performed through nonlinear registration with antsRegistration (ANTs 2.5.0), using brain-extracted versions of both T1w reference and the T1w template. The following template was selected for spatial normalization and accessed with TemplateFlow (23.1.0, Ciric et al., 2022): ICBM 152 Nonlinear Asymmetrical template version 2009c [Fonov et al. (2009), RRID:SCR_008796; TemplateFlow ID: MNI152NLin2009cAsym].

***Functional data preprocessing***

For each of the 2 BOLD runs found per subject (across all tasks and sessions), the following preprocessing was performed. First, a reference volume was generated from the shortest echo of the BOLD run, using a custom methodology of fMRIPrep, for use in head motion correction. Head-motion parameters with respect to the BOLD reference (transformation matrices, and six corresponding rotation and translation parameters) are estimated before any spatiotemporal filtering using MCFLIRT (FSL, Jenkinson et al., 2002). The BOLD reference was then co-registered to the T1w reference using bbregister (FreeSurfer) which implements boundary-based registration (Greve & Fischl, 2009). Co-registration was configured with six degrees of freedom. Several confounding time-series were calculated based on the preprocessed BOLD: framewise displacement (FD), DVARS and three region-wise global signals. FD was computed using two formulations following Power (absolute sum of relative motions (Power et al., 2014)) and Jenkinson (relative root mean square displacement between affines, (Jenkinson et al., 2002)). FD and DVARS are calculated for each functional run, both using their implementations in Nipype (following the definitions by Power et al. (2014)). The three global signals are extracted within the CSF, the WM, and the whole-brain masks. Additionally, a set of physiological regressors were extracted to allow for component-based noise correction (CompCor, Behzadi et al., 2007). Principal components are estimated after high-pass filtering the preprocessed BOLD time-series (using a discrete cosine filter with 128s cut-off) for the two CompCor variants: temporal (tCompCor) and anatomical (aCompCor). tCompCor components are then calculated from the top 2% variable voxels within the brain mask. For aCompCor, three probabilistic masks (CSF, WM and combined CSF+WM) are generated in anatomical space. The implementation differs from that of Behzadi et al. in that instead of eroding the masks by 2 pixels on BOLD space, a mask of pixels that likely contain a volume fraction of GM is subtracted from the aCompCor masks. This mask is obtained by dilating a GM mask extracted from the FreeSurfer’s aseg segmentation, and it ensures components are not extracted from voxels containing a minimal fraction of GM. Finally, these masks are resampled into BOLD space and binarized by thresholding at 0.99 (as in the original implementation). Components are also calculated separately within the WM and CSF masks. For each CompCor decomposition, the k components with the largest singular values are retained, such that the retained components’ time series are sufficient to explain 50 percent of variance across the nuisance mask (CSF, WM, combined, or temporal). The remaining components are dropped from consideration. The head-motion estimates calculated in the correction step were also placed within the corresponding confounds file. The confound time series derived from head motion estimates and global signals were expanded with the inclusion of temporal derivatives and quadratic terms for each (Satterthwaite et al., 2013). Frames that exceeded a threshold of 0.9 mm FD or 1.5 standardized DVARS were annotated as motion outliers. Additional nuisance timeseries are calculated by means of principal components analysis of the signal found within a thin band (crown) of voxels around the edge of the brain (Patriat et al., 2017). All resamplings can be performed with a single interpolation step by composing all the pertinent transformations (i.e., head-motion transform matrices, susceptibility distortion correction when available, and co-registrations to anatomical and output spaces). Gridded (volumetric) resamplings were performed using nitransforms, configured with cubic B-spline interpolation.

Many internal operations of fMRIPrep use Nilearn 0.10.2 (Abraham et al., 2014; RRID:SCR_001362), mostly within the functional processing workflow. For more details of the pipeline, see the section corresponding to workflows in fMRIPrep’s documentation.

The above boilerplate text was automatically generated by fMRIPrep with the express intention that users should copy and paste this text into their manuscripts unchanged. It is released under the CC0 license. Although not included in the boilerplate, our fMRIprep pipeline also employed the optimally weighted combination of the multi-echo data using tedana (Ahmed et al., 2023).

Supplementary Table 1: Left - The 17 independent components (ICs) derived from our group-ICA analysis along with their network labels. Right - The intrinsic-connectivity networks (ICNs) from Laird et al. (2011), as well as the r-values from our cross correlation analysis via fslcc. All r-values greater than 0.3 are shown. Artifactual ICs were not included.

| ICs from Present Study (network label) | Associated highest ICN from Laird et al. (r-value) |
| --- | --- |
| 1 (Higher Visual Network) | ICN 11 (r = 0.74)  ICN 10 (r = 0.35) |
| 2 (Attention Control Network 1) | ICN 10 (r = 0.45)  ICN 7 (r = 0.36) |
| 4 (Visual Network) | ICN 12 (r = 0.80) |
| 5 (Default Mode Network 1) | ICN 13 (r = 0.42) |
| 6 (Left Frontoparietal Network) | ICN 18 (r = 0.56) |
| 7 (Salience Network) | ICN 4 (r = 0.53)  ICN 3 (r = 0.30) |
| 8 (Sensorimotor Language Network) | ICN 17 (r = 0.62) |
| 9 (Default Mode Network 3) | ICN 13 (r = 0.37)  ICN 16 (r = 0.34) |
| 10 (Default Mode Network 2) | ICN 13 (r = 0.48) |
| 11 (Somatosensory Network 1) | ICN 8 (r = 0.60) |
| 12 (Somatosensory Network 2) | ICN 9 (r = 0.63) |
| 13 (Attention Control Network 2) | ICN 7 (r = 0.37) |
| 14 (Right Frontoparietal Network) | ICN 15 (r = 0.59) |
| 15 (Reward Network 2) | ICN 2 (r = 0.47) |
| 17 (Limbic Network) | ICN 1 (r = 0.45) |
| 18 (Reward Network 1) | ICN 1 (r = 0.46) |
| 20 (Cerebellum Basal Ganglia Network) | ICN 14 (r = 0.70)  ICN 5 (r = 0.37) |
